# Single nucleotide replacement in the Atlantic salmon genome using CRISPR/Cas9 and asymmetrical oligonucleotide donors

**DOI:** 10.1186/s12864-021-07823-8

**Published:** 2021-07-22

**Authors:** Anne Hege Straume, Erik Kjærner-Semb, Kai Ove Skaftnesmo, Hilal Güralp, Simon Lillico, Anna Wargelius, Rolf Brudvik Edvardsen

**Affiliations:** 1 Institute of Marine Research, P.O. Box 1870, Nordnes, NO-5817 Bergen, Norway; 2 The Roslin Institute and R(D)SVS, University of Edinburgh, Easter Bush Campus, Midlothian, EH25 9RG UK

**Keywords:** HDR, ODN, Gene editing, Knock-in, Aquaculture, New breeding technologies

## Abstract

**Background:**

New breeding technologies (NBT) using CRISPR/Cas9-induced homology directed repair (HDR) has the potential to expedite genetic improvement in aquaculture. The long generation time in Atlantic salmon makes breeding an unattractive solution to obtain homozygous mutants and improving the rates of perfect HDR in founder (F0) fish is thus required. Genome editing can represent small DNA changes down to single nucleotide replacements (SNR). This enables edits such as premature stop codons or single amino acid changes and may be used to obtain fish with traits favorable to aquaculture, e.g. disease resistance. A method for SNR has not yet been demonstrated in salmon.

**Results:**

Using CRISPR/Cas9 and asymmetrical ODNs, we were able to perform precise SNR and introduce a premature stop codon in *dnd* in F0 salmon. Deep sequencing demonstrated up to 59.2% efficiency in single embryos. In addition, using the same asymmetrical ODN design, we inserted a FLAG element into *slc45a2* and *dnd*, showing high individual perfect HDR efficiencies (up to 36.7 and 32.7%, respectively).

**Conclusions:**

In this work, we demonstrate that precise SNR and knock-in (KI) can be performed in F0 salmon embryos using asymmetrical oligonucleotide (ODN) donors. We suggest that HDR-induced SNR can be applied as a powerful NBT, allowing efficient introgression of favorable alleles and bypassing challenges associated with traditional selective breeding.

**Supplementary Information:**

The online version contains supplementary material available at 10.1186/s12864-021-07823-8.

## Background

There is an increasing demand for sustainable animal husbandry, and the fast-growing fish aquaculture industry is a food production sector with great potential to improve global food security. Fish aquaculture is also considered to be efficient in terms of feed conversion and protein retention compared to most terrestrial livestock [1, 2]. Atlantic salmon (*Salmo salar* L.) is farmed in the sea at a large scale, but further growth is currently hindered by a range of issues including genetic introgression of escapees into wild populations and the spread of disease [3, 4]. New breeding technologies (NBT) using gene editing offer an exciting opportunity to increase the sustainability of open sea-cage salmon farming by allowing us to induce both sterility and disease resistance [2, 5–7].

A CRISPR/Cas9 induced double-stranded DNA break (DSB) in the coding sequence of a gene, followed by activation of the endogenous non-homologous end joining (NHEJ) pathway, results in an array of unpredictable insertions or deletions that may result in frameshift and gene knock-out (KO). This is a useful approach to study KO phenotypes, and has been applied successfully in salmon [5, 7, 8] and several other farmed fish species [9–24]. To make precise genome alterations, it is a necessity to induce homology directed repair (HDR) by supplying a repair template homologous to the CRISPR target site, thereby allowing to change SNPs, insert affinity tags for protein detection and modify regulatory elements to alter expression of target genes. An example is channel catfish, where HDR mediated editing was used to increase disease resistance [25]. A single nucleotide replacement (SNR) can be used to introduce favorable wild type alleles and could be a promising and time saving solution compared to traditional breeding with backcrossing and selection. The genetic progress in selective breeding programs is also limited by the heritability of the target traits, and the standing genetic variation in the broodstock. NBT using CRISPR/Cas9-induced HDR can offer new solutions and opportunities in these areas [2, 26]. An important issue when it comes to gene editing in salmon, is to reduce mosaicism in individual founder (F0) fish. The long generation time (3–4 years) makes breeding an unattractive option to obtain homozygous mutants, and most functional studies must be performed in F0. Thus, improving the efficiency of perfect editing in F0 individuals is crucial.

We have previously demonstrated highly efficient HDR in salmon using symmetrical oligonucleotides (ODNs) with short (24/48/84 bp) homology arms to knock-in (KI) a FLAG element in the pigmentation gene *solute carrier family 45 member 2* (*slc45a2).* Using high-throughput sequencing (HTS), we showed in vivo ODN-mediated KI in almost all the gene edited animals and demonstrated perfect HDR integration rates of up to 27% in individual F0 embryos [27]. Short homology arms have also been shown to induce efficient HDR in zebrafish [28, 29].

In this work, we aimed to perform SNR and increase the rates of perfect HDR in individual F0 salmon. Asymmetrical ODNs in combination with CRISPR/Cas9 have previously been demonstrated to improve HDR rates in cell cultures [30] and induced pluripotent stem cells [31]. Using asymmetrical ODNs, we have successfully performed SNR and introduced a premature stop codon in the primordial germ cell survival factor gene *dead end* (*dnd*). In addition, also using asymmetrical ODNs, we have inserted FLAG elements into both *slc45a2* and *dnd*. SNR was more efficient than FLAG KI, suggesting that HDR efficiency may be inversely proportional with insert size. As previously [27], we found HDR efficiency to be dependent on template concentration, but suggest using the lowest possible concentration to avoid toxicity and enable targeting multiple genes at the same time. Our results show that CRISPR/Cas9 in combination with asymmetrical ODNs enables rapid and precise changes to the genome in individual F0 animals and present a promising tool for fish breeders in the future.

## Results

### FLAG KI targeting *slc45a2* and *dnd*

**Fig. 1:**
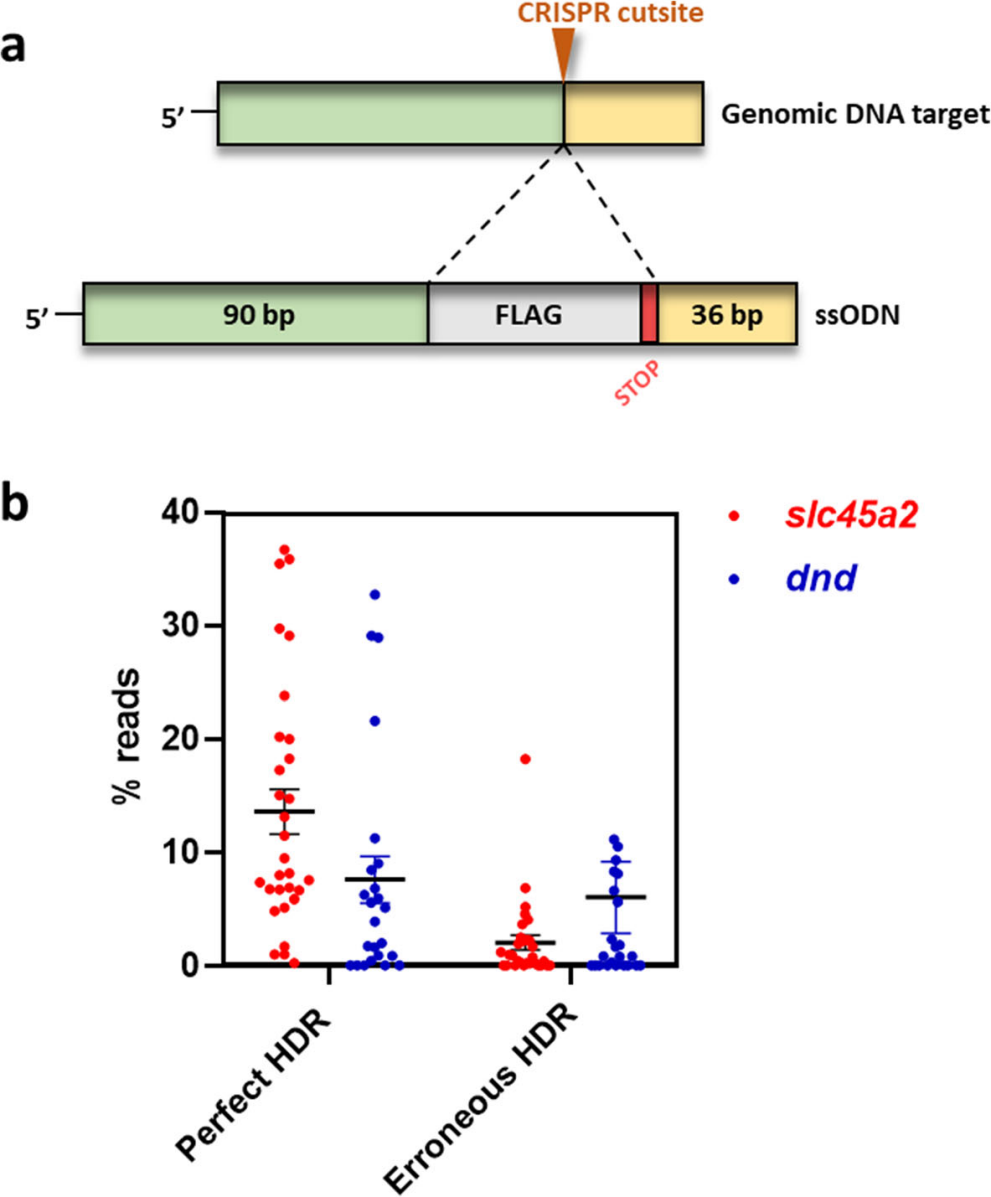
*slc45a2* and *dnd* FLAG knock-in (1.5 μM ODN). **a.** Asymmetrical ODNs were designed by copying 90 + 36 nucleotides on each side of the CRISPR cut site flanking the insert (indicated with a dotted line) containing the FLAG element followed by a STOP codon (TAA). **b.** Relative read counts per individual for *slc45a2* (red dots, *n* = 30) and *dnd* (blue dots, *n* = 24). Reads with a perfect match to the entire target sequence are referred to as perfect HDR. Reads with a correct insert flanked by mismatches/indels on the 5′ and/or 3′-side are referred to as erroneous HDR. Error bars indicate SEM/group

Targeting *slc45a2* [5] and *dnd* [7], we have here performed KI of a FLAG element in F0 salmon using CRISPR/Cas9 and asymmetrical ODNs (Fig. 1a and Fig. S1). Analyzing the rate of perfect HDR in individual animals by HTS of amplicons, we detected an average of 13.6% (std 10.9%) for *slc45a2* and 7.6% (std 10.1%) for *dnd* (Fig. 1b). Interestingly, individuals in both groups displayed a very high efficiency with up to 36.7% perfect HDR in *slc45a2*, and 32.7% in *dnd* (Table S1). This is higher than our previously reported results, using symmetrical ODNs at 1.5 μM [27]. When comparing the efficiency of FLAG KI (targeting *slc45a2*) using asymmetrical ODNs described herein, to symmetrical ODNs described before [27], a significant difference (*P* < 0.0001) was detected for average perfect HDR between symmetrical (5.1%) and asymmetrical ODNs (13.6%). No significant difference was detected when comparing the average rates of erroneous HDR between symmetrical (3.1%) and asymmetrical ODNs (2.0%) (Fig. S2).

### Oligonucleotide concentration affects HDR efficiency

**Fig. 2:**
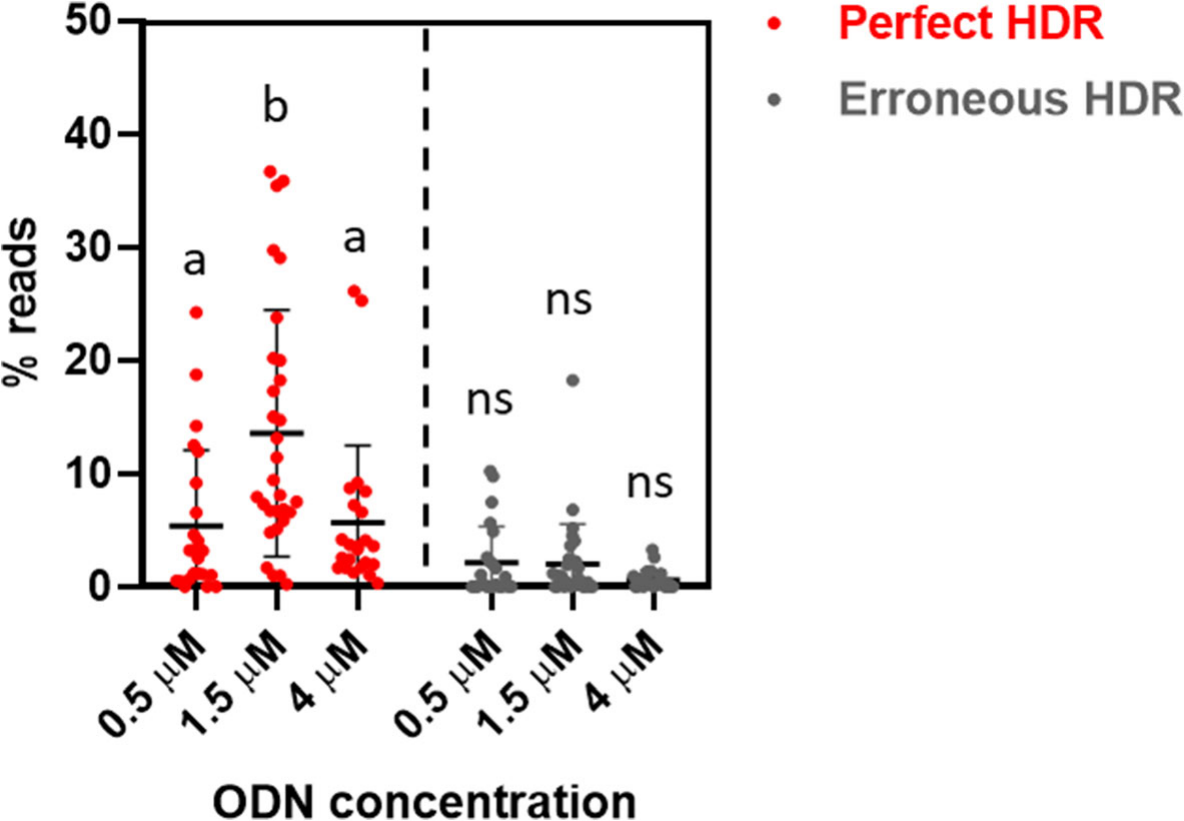
*slc45a2* FLAG knock-in. The asymmetrical ODN targeting *slc45a2* was tested using three different concentrations: 0.5 (*n* = 23), 1.5 (*n* = 30) and 4.0 (*n* = 23) μM. Sequence reads with a perfect match to the entire target sequence are referred to as perfect HDR and reads with a correct insert but mismatches/indels in the homology arms are referred to as erroneous HDR. Read counts for each sample are given in % of the total number of reads. The error bars indicate SEM/group. Different lowercase letters indicate significant differences (*P* < 0.05), ns = non-significant

We and others [27, 32] have shown that increasing the concentration of the DNA donor improves HDR efficiency. However, DNA can be toxic to cells and we wanted to elucidate if there is a trade-off between high integration efficiency and toxicity by testing the *slc45a2* FLAG KI ODN at three different concentrations: 0.5, 1.5 and 4.0 μM (Fig. 2). In accordance with our previous results, a template concentration of 1.5 μM resulted in the most efficient KI. We detected the approximately same average efficiency when using 0.5 and 4 μM. However, the highest concentration resulted in fewer pure albinos and a higher degree of mosaicism compared to individuals injected with lower concentrations of template (Fig. S3). As expected, the HTS results from the animals who had received the highest dose revealed a much higher percentage of wild type reads (Table S1).

### Asymmetrical ODNs induce highly efficient single nucleotide replacement

**Fig. 3:**
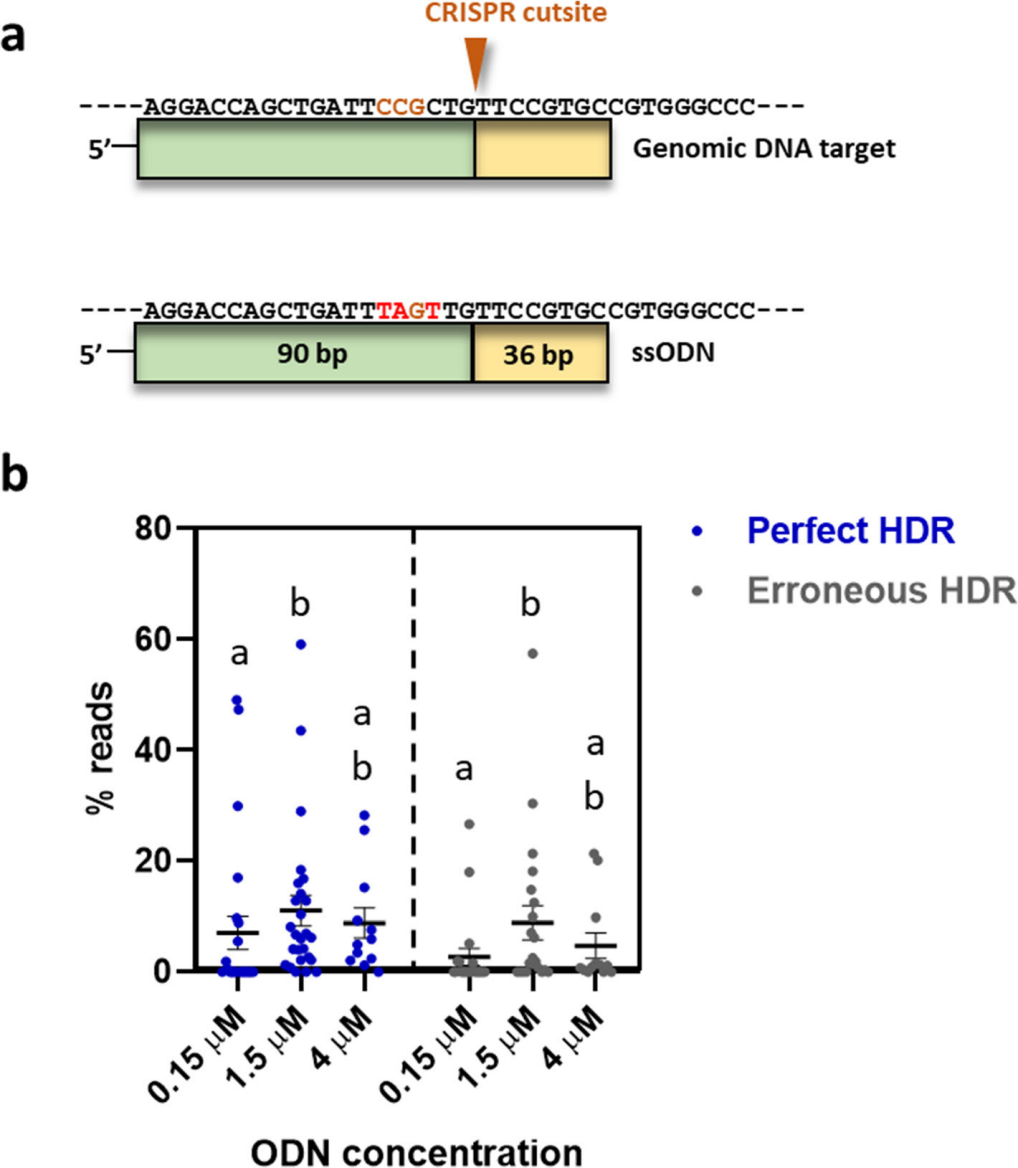
Single nucleotide replacement in *dnd*. **a.** An asymmetrical ODN targeting *dnd* was designed with 90 + 36 nucleotides on each side of the CRISPR cut site and three nucleotides were changed. PAM site is shown with brown letters, and novel nucleotides with red letters. **b.** HDR rates for three different ODN concentrations; 0.15 (*n* = 24), 1.5 (*n* = 26) and 4.0 μM (*n* = 12). Sequence reads with a perfect match to the entire target sequence are referred to as perfect HDR (blue) and reads with a correct SNR but mismatches/indels in the homology arms are referred to as erroneous HDR (gray). Read counts for each sample are given in % of the total number of reads. Error bars indicate SEM/group. Different lowercase letters indicate significant differences (*P* < 0.05)

Targeting *dnd*, we performed a SNR using an asymmetrical ODN while at the same time continuing to refine the ODN concentration. Using 0.15, 1.5 and 4 μM ODN concentrations, we obtained an average perfect HDR of 7.4% (std 14.8), 12.5% (std 14.3) and 7.4% (std 9.4), respectively (Fig. 3). However, when analyzing individual fish, the most striking result was obtained using 1.5 μM, in which perfect repair efficiency of up to 59.2% was detected. To our knowledge, this level of perfect HDR in F0 has not been reported in any other fish. Even at the lowest concentration (0.15 μM), two individuals displayed 49.1 and 47.4% perfect HDR.

### ODN polarity affects indel locations

In addition to reads displaying perfect HDR, we detected reads displaying erroneous repair, meaning reads with a correct FLAG-insert/SNR but also indels on the 5′- and/or 3′-side of the insert/SNR (Figs. 1, 2 and 3b).

**Fig. 4:**
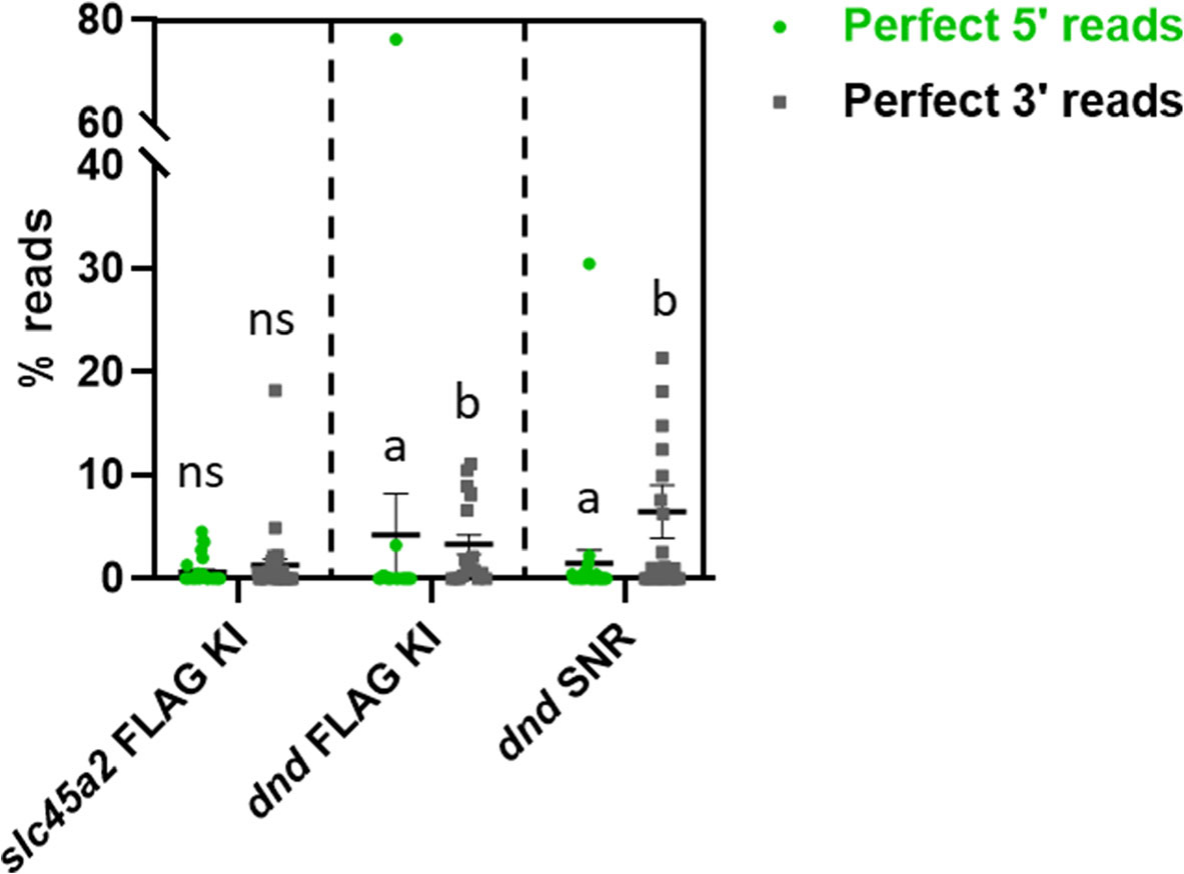
Variation in indel locations. Here, we distinguished between reads with a perfect match to the 5′- or 3′-side of the FLAG insert/SNR. The asymmetrical ODNs were compared at 1.5 μM. Green dots represent perfect 5′ reads and squares represent perfect 3′ reads. Read counts for each sample are given in % of the total number of reads. Error bars indicate SEM/group. The groups *slc45a2* FLAG KI (*n* = 30), *dnd* FLAG KI (*n* = 19) and *dnd* SNR (*n* = 24) were analyzed separately. Different lowercase letters indicate significant differences (*P* < 0.05)

In our previous work using symmetrical ODNs, we revealed a strong correlation between ODN polarity and the location of these indels on either the 5′- or 3′-side of the inserted sequence. According to this, when using a repair template with sense orientation relative to the target strand, most of the indels will end up on the 5′-side of the insert [27]. In the current study the asymmetrical ODNs were sense relative to the target strand (Fig. S1a and b), and we observed that most of the indels were located on the 5′-side of the insert (fewer reads with perfect 5′-reads than 3′-reads), supporting our previous findings. A significant difference was detected for *dnd* KI (*P* = 0.034) and SNR (*P* < 0.029) and non-significant for *slc45a2* KI (Fig. 4).

## Discussion

This study demonstrated that asymmetrical ODNs induce efficient and precise HDR in salmon. Importantly, they appear to be more efficient than symmetrical ODNs, as compared to our previous results [27] (Fig. S2). No significant difference was detected when comparing the efficiency of FLAGKI in *slc45a2* and *dnd* (Fig. 1). This finding suggests that HDR may be applied successfully to any gene of interest.

For the first time in salmon, we performed a successful SNR by introducing a premature stop codon to the *dnd* gene. Compared to FLAGKI we found SNR to be the most efficient approach. We speculate that the high individual HDR efficiency obtained for SNR is due to the lack of insert, as editing efficiency has been shown to be sensitive to insert size [33]. For some genes and applications, it may not be relevant to use HDR to insert DNA, but rather to obtain SNR which can translate into for example single amino acid changes, or like here, premature stop codon formation. One such example is the *vgll3* gene which contains two missense SNPs, resulting in either early or late maturity in Atlantic salmon [3]. Developing precise gene editing technology to make such small edits may therefore be a useful tool in NBT, enabling introgression of natural beneficial variants into aquaculture strains. When CRISPR/Cas9 is used to make a traditional KO through NHEJ, one of the challenges is that the mutation can be in-frame and therefore potentially silent. SNR could solve this by insertion of a novel stop codon, and as such increase the levels of functional KO mutations by decreasing the chance of in-frame indels.

Although we found HDR efficiency to be dependent on template concentration, several parameters may contribute to this, such as fluctuation in temperature and variable presence of parasites (mostly *Saprolegnia parasitica*) between tanks. This often results in variable lethality, unrelated to injections as this is observed also in non-injected groups. Also, during the microinjection procedure there will be inevitable variation in the volume injected into each fertilized egg. Performing precise microinjections by hand can be challenging due to the opaque salmon eggs, and personal skills will influence the outcome. Technical aspects will also matter, such as variation in the diameter of the needle opening and the egg quality. When sampling the best phenotypic albino individuals there were visible difference in mosaicism between the groups injected with low (0.15 / 0.5 μM) and high (4 μM) template concentration (Fig. S3). This was true for both the *slc45a2* and *dnd* experiments. It is therefore conceivable that the mosaicism observed for the high dose group (4 μM ODN) is due to toxicity of the injection mix when the injected volume is high. We hypothesize that the surviving eggs received a lower volume of the injection mix, and therefore also a lower dose of the *cas9* and guide RNA resulting in a more mosaic phenotype. Taking this into account, it could be an advantage to use the lowest possible ODN concentration to avoid unnecessary DNA induced toxicity, which would also allow editing multiple genes at the same time.

## Conclusion

We show that it is possible to use CRISPR/Cas9-induced HDR in NBT to obtain desirable traits. SNR is a promising tool to insert favorable alleles in farmed salmon and, considering the long generation time, more convenient than crossing in traits through conventional breeding. Moreover, this could also be an advantage for aquaculture species in general (e.g trout, sea bass, tilapia). This technology offers an exciting opportunity to insert traits of interest into the recently demonstrated fertile but genetically sterile salmon [26]. This fish will produce sterile offspring and may therefore represent the future salmon aquaculture by combining sterility and other favorable traits induced by HDR, such as disease resistance.

## Methods

### Preparation of Cas9 RNA, gRNAs and ODNs

The CRISPR target sequences for *slc45a2* and *dnd* are described in Edvardsen et al. [5], and Wargelius et al. [7], respectively. Preparation of gRNAs and *cas9* mRNA was performed as previously described [5, 27]. The RNeasy MiniKit spin column (Qiagen) was used to purify the gRNA. The ODNs were ordered from Integrated DNA Technologies (Leuven, Belgium). The ODN design is based on Richardson et al. [30]

### Microinjection

Salmon eggs and sperm were delivered by Mowi (Hauglandshella, Askøy, Norway). Fertilization and microinjections were carried out as described previously [5] using 50 ng/μl gRNA and 150 ng/μl *cas9* mRNA in nuclease free water and a FemtoJet®4i (Eppendorf) microinjector. The ODNs were added to the injection mix with a final concentration of 0.15, 0.5, 1.5 or 4 μM. The selection of ODN concentrations were based on Straume et al. [27] and Boel et al. [28] Non-injected embryos were used as controls for all experiments.

### Analysis of mutants

As described previously [27] *slc45a2* mutants were selected based on visual inspection of newly hatched larvae (~ 680 day degrees). When editing *dnd*, we also added the *slc45a2* gRNA to the injection mix to obtain a visual phenotype, and thus make it easier to select the mutants. DNA was extracted from caudal fins using DNeasy Blood & Tissue kit (Qiagen). DNA extracted from the fin has previously been shown to be broadly representative for the whole fish [5, 26]. A fragment covering the entire CRISPR target sites for *slc45a2* and *dnd* was amplified with a two-step fusion PCR (as described in Gagnon et.al 2014) to prepare for Illumina MiSeq. The following primers (gene specific sequence indicated in capital letters) were used in the first PCR-step for *slc45a2*:

5′-tctttccctacacgacgctcttccgatctCAGATGTCCAGAGGCTGCTGCT and.

5′-tggagttcagacgtgtgctcttccgatctTGCCACAGCCTCAGAATGTACA. The following primers (gene specific sequence indicated in capital letters) were used in the first PCR-step for *dnd*:

5′-tctttccctacacgacgctcttccgatctGGGGAAAGGCTAGGGAGAGA and.

5′-tggagttcagacgtgtgctcttccgatct CGGTTCTGTCCGCTGAAGTT.

### Analysis of MiSeq data

Read counts were reported for variants containing the inserted or edited sequence, separating those with a perfect match to the entire target sequence (referred to as perfect HDR), and those with a correct insert sequence/SE, but mismatches in the rest of the target sequence (referred to as erroneous HDR). In addition, read counts were reported for wild type sequences.

The settings applied for filtering, trimming and variant calling of the MiSeq reads are illustrated in Fig. S4, and described below:

Fastq files were filtered and trimmed with the following specifications; primer sequences were used to demultiplex reads from different amplicons on the same sequencing run, minimum read length was set to 100 bp, and forward and reverse reads were assembled to correct sequencing errors (minimum overlap between forward and reverse reads was set to 150 bp for *slc45a2* and 200 bp for *dnd*, and allowing maximum 20% mismatches between forward and reverse reads in the overlap region). Assembled reads were combined with forward reads that did not pass the assembly thresholds. Variants were then called using positions 20–200 for *slc45a2* and positions 60–230 for *dnd*. All bases with base quality < 20 were converted to N’s, and maximum 5 N’s were allowed per read. Identical reads were then grouped (referred to as variants), and variants that only differed by up to 5 N’s were grouped if none of the variants differed by any nucleotides. For each group, the variant with the least N’s was chosen as representative. We only retained variants supported by a minimum of 100 reads and variants were grouped if they differed by up to 5 N’s if none of the variants differed by any nucleotides.

### Statistical analyses

D’Agostino Person normality test (column statistics) were used to assess normal distribution of the data. None of the groups displayed normal distribution, and we carried on with non-parametric analyses. When analyzing more than two groups, non-parametric statistical analyses were performed using a Kruskall-Wallis test, followed by Dunn’s multiple comparison test. When analyzing two groups, a Mann-Whitney rank test, or a Wilcoxon paired test was performed. The tests were carried out using GraphPad Prism 8.0.1.

## Supplementary Information

**Additional file 1: Table S1. **Individual HDR efficiency.

**Additional file 2: Figure S1.** a Asymmetrical ODN design *slc45a2.* Fig. S1b Asymmetrical ODN design *dnd.*

**Additional file 3: Figure S2.** Comparison of symmetrical and asymmetrical ODNs for *slc45a2* FLAG knock-in. All the ODNs compared here were designed for *slc45a2* to KI a FLAG element. The ODN concentration was 1.5 μM. The symmetrical ODNs are a pool of S 24, AS 24, ds 24, S 48 and S 84 (described in Straume et.al 2020). The asymmetrical ODN design is illustrated in Suplemmentary Fig. 1 A. Green dots represent perfect HDR, black squares represent erroneous HDR. Mutant fish were analysed using Illumina MiSeq. Read counts for each sample are given in % of the total number of reads with at least 100 identical reads. The error bars indicate the SEM of the mean for each group. A Mann-Whitney test was used to compare the mean rank of symmetrical vs. asymmetrical ODNs, analyzing the groups perfect and erroneous HDR separately. Different lower-case letters indicate significant differences (*P* < 0.05).

**Additional file 4: Figure S3**. Example of fry sampling based on visual inspection of pigmentation. Fig. S3 a *slc45a2* FLAG KI (0.5 μM). Fig. S3 b *slc45a2* FLAG KI (4 μM). Fig. S3 c *dnd* SNR (0.15 μM). Fig. S3 d *dnd* SNR (4 μM). *: wild type.

**Additional file 5: Figure S4.** Illustration of the settings applied for filtering, trimming and variant calling of the MiSeq reads. Fastq files were filtered and trimmed with the following specifications; primer sequences were used to demultiplex reads from different amplicons on the same sequencing run, minimum read length was set to 100 bp, and forward and reverse reads were assembled to correct sequencing errors (minimum overlap between forward and reverse reads was set to 150 bp for *slc45a2* and 200 bp for *dnd* and allowing at most 20% mismatches between forward and reverse reads in the overlap region). Assembled reads were combined with forward reads that did not pass the assembly thresholds. Variants were then called using positions 20–200 for *slc45a2* and positions 60–230 for *dnd*. All bases with base quality < 20 were converted to N’s, and maximum 5 N’s were allowed per read. Identical reads were then grouped (referred to as variants) → variants that were only differing by up to 5 N’s were grouped if none of the variants differed by any nucleotides → for each group the variant with the least N’s was chosen as representative → only retained variants supported by a minimum of 100 reads → variants were grouped if they differed by up to 5 N’s if none of the variants differed by any nucleotides. Finally, read counts were reported for the variants containing the inserted or edited sequence, separating those with a perfect match to the entire target sequence, and those with a correct insert sequence, but mismatches in the rest of the target sequence. In addition, read counts were reported wild type sequences.

## Data Availability

Data generated or analyzed during this study are included in this article (and its Supplementary Information).
