# Supplementary figures and images for: Single nucleotide replacement in the Atlantic salmon genome using CRISPR/Cas9 and asymmetrical oligonucleotide donors
Source: BMC Genomics. 2021 Jul 22;22:563. doi: 10.1186/s12864-021-07823-8 (PMC8296724; doi:10.1186/s12864-021-07823-8)

## Slide 1
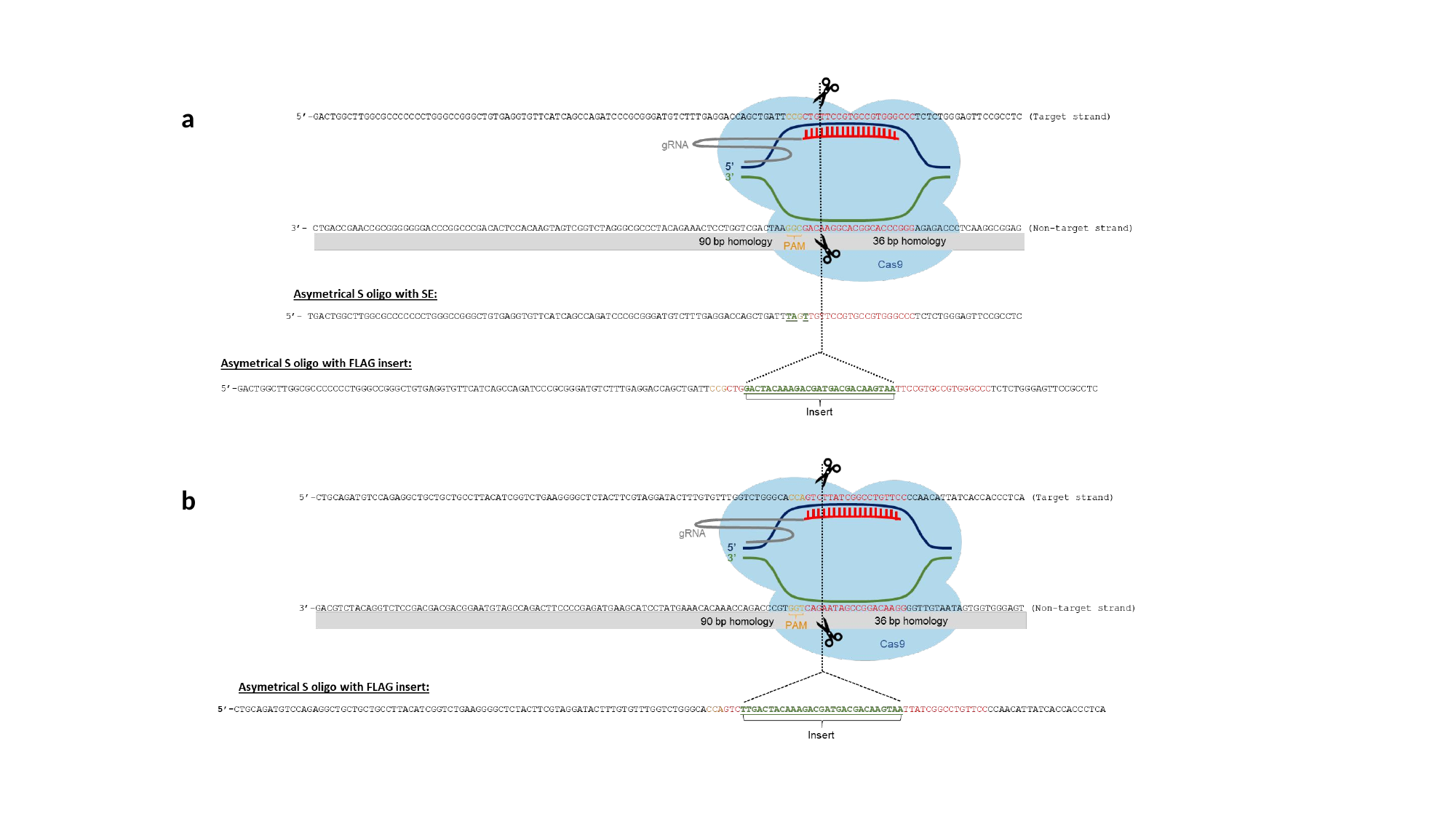

a
b

Supplement: Supplementary file 2 — Additional file 2: Figure S1. a Asymmetrical ODN design slc45a2. Fig. S1b Asymmetrical ODN design dnd. [file 12864_2021_7823_MOESM2_ESM.pptx]

## Slide 1
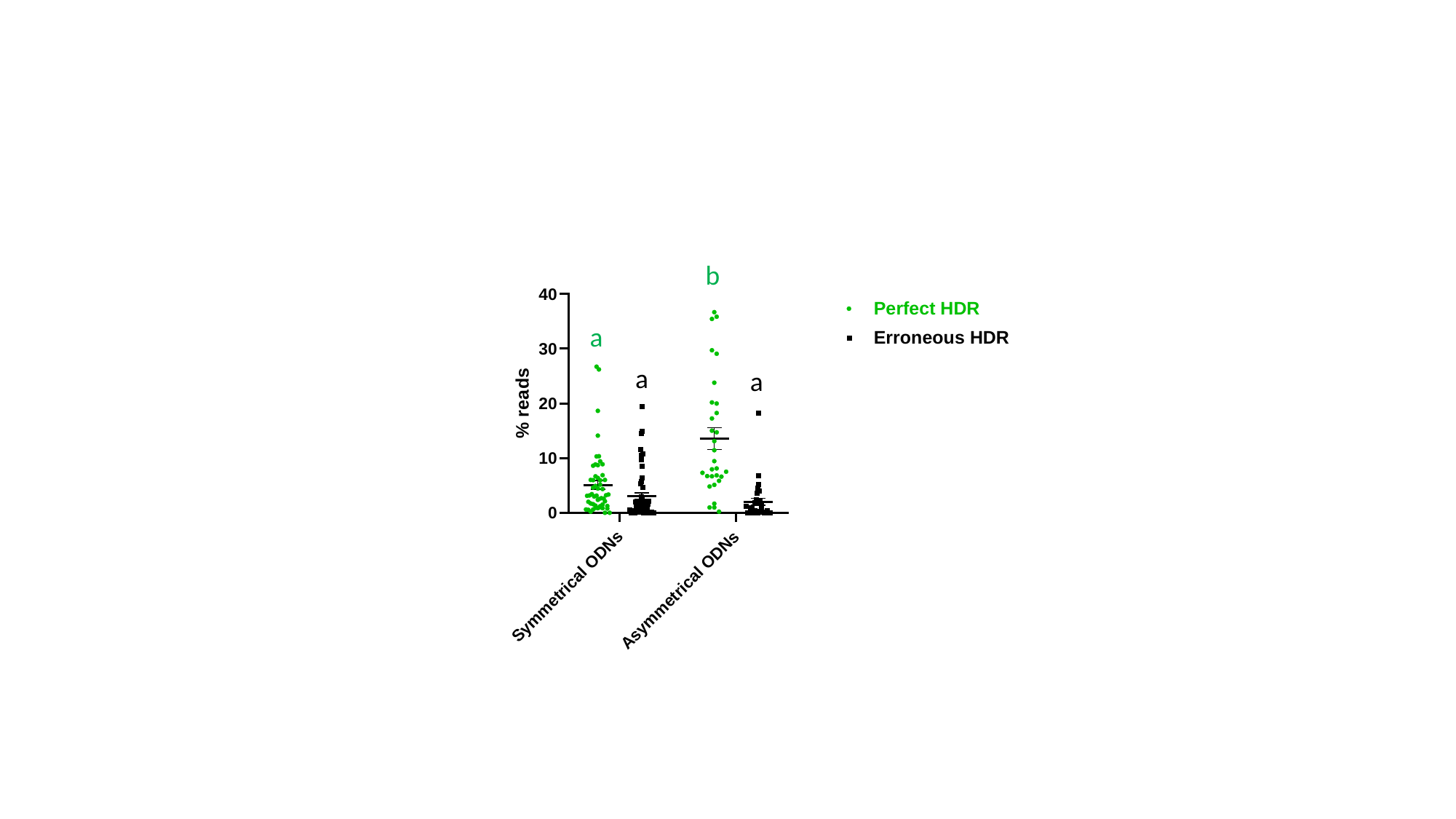

b
a
a
a

Supplement: Supplementary file 3 — Additional file 3: Figure S2. Comparison of symmetrical and asymmetrical ODNs for slc45a2 FLAG knock-in. All the ODNs compared here were designed for slc45a2 to KI a FLAG element. The ODN concentration was 1.5 μM. The symmetrical ODNs are a pool of S 24, AS 24, ds 24, S 48 and S 84 (described in Straume et.al 2020). The asymmetrical ODN design is illustrated in Suplemmentary Fig. 1 A. Green dots represent perfect HDR, black squares represent erroneous HDR. Mutant fish were analysed using Illumina MiSeq. Read counts for each sample are given in % of the total number of reads with at least 100 identical reads. The error bars indicate the SEM of the mean for each group. A Mann-Whitney test was used to compare the mean rank of symmetrical vs. asymmetrical ODNs, analyzing the groups perfect and erroneous HDR separately. Different lower-case letters indicate significant differences (P < 0.05). [file 12864_2021_7823_MOESM3_ESM.pptx]

## Slide 1
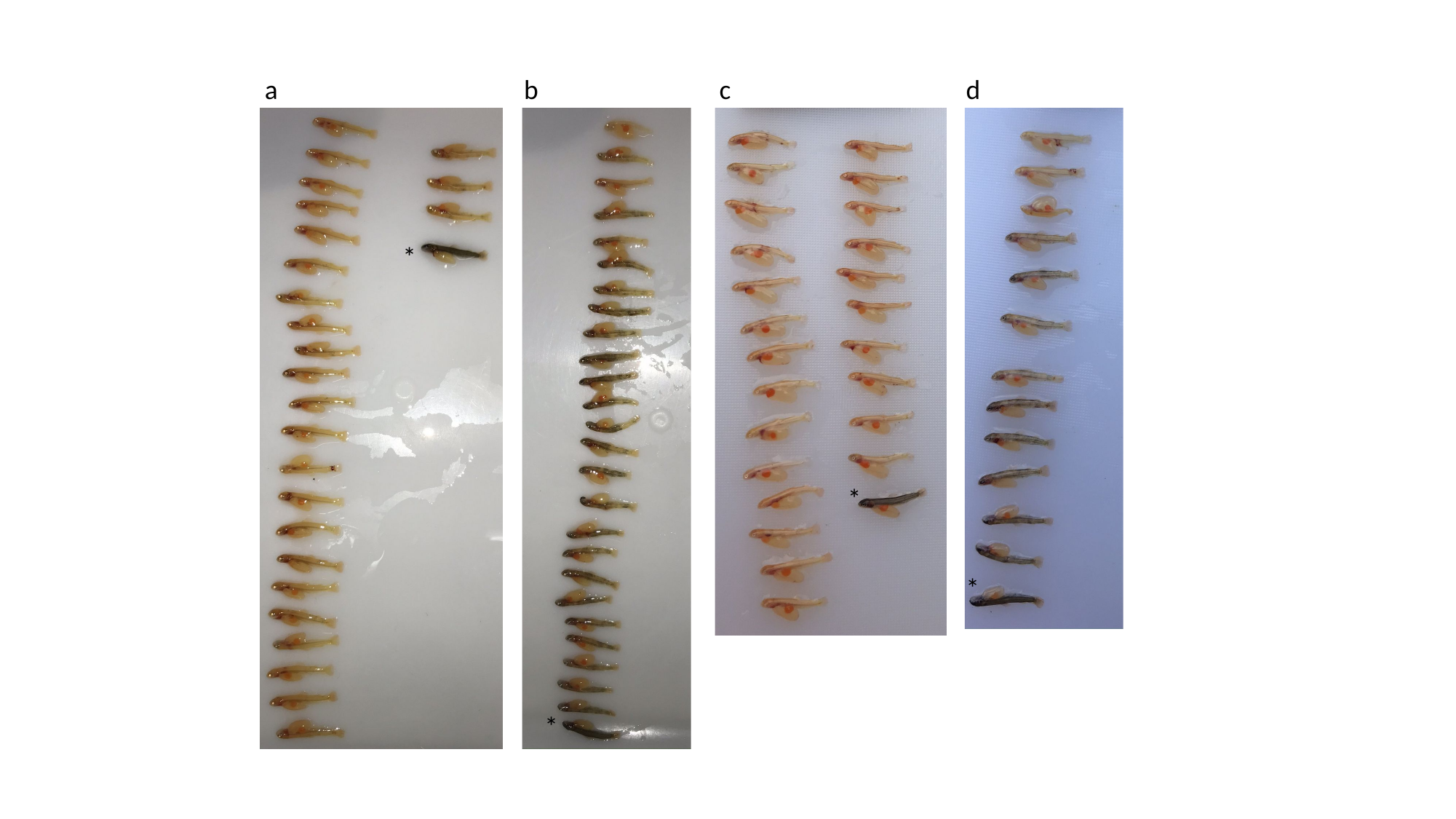

a
b
c
d
*
*
*
*

Supplement: Supplementary file 4 — Additional file 4: Figure S3. Example of fry sampling based on visual inspection of pigmentation. Fig. S3 a slc45a2 FLAG KI (0.5 μM). Fig. S3 b slc45a2 FLAG KI (4 μM). Fig. S3 c dnd SNR (0.15 μM). Fig. S3 d dnd SNR (4 μM). *: wild type. [file 12864_2021_7823_MOESM4_ESM.pptx]
