# Supplementary material for: Single nucleotide replacement in the Atlantic salmon genome using CRISPR/Cas9 and asymmetrical oligonucleotide donors
Source: BMC Genomics. 2021 Jul 22;22:563. doi: 10.1186/s12864-021-07823-8 (PMC8296724; doi:10.1186/s12864-021-07823-8)
Supplement: Supplementary file 5 — Additional file 5: Figure S4. Illustration of the settings applied for filtering, trimming and variant calling of the MiSeq reads. Fastq files were filtered and trimmed with the following specifications; primer sequences were used to demultiplex reads from different amplicons on the same sequencing run, minimum read length was set to 100 bp, and forward and reverse reads were assembled to correct sequencing errors (minimum overlap between forward and reverse reads was set to 150 bp for slc45a2 and 200 bp for dnd and allowing at most 20% mismatches between forward and reverse reads in the overlap region). Assembled reads were combined with forward reads that did not pass the assembly thresholds. Variants were then called using positions 20–200 for slc45a2 and positions 60–230 for dnd. All bases with base quality < 20 were converted to N’s, and maximum 5 N’s were allowed per read. Identical reads were then grouped (referred to as variants) → variants that were only differing by up to 5 N’s were grouped if none of the variants differed by any nucleotides → for each group the variant with the least N’s was chosen as representative → only retained variants supported by a minimum of 100 reads → variants were grouped if they differed by up to 5 N’s if none of the variants differed by any nucleotides. Finally, read counts were reported for the variants containing the inserted or edited sequence, separating those with a perfect match to the entire target sequence, and those with a correct insert sequence, but mismatches in the rest of the target sequence. In addition, read counts were reported wild type sequences. [file 12864_2021_7823_MOESM5_ESM.pptx]

## Slide 1
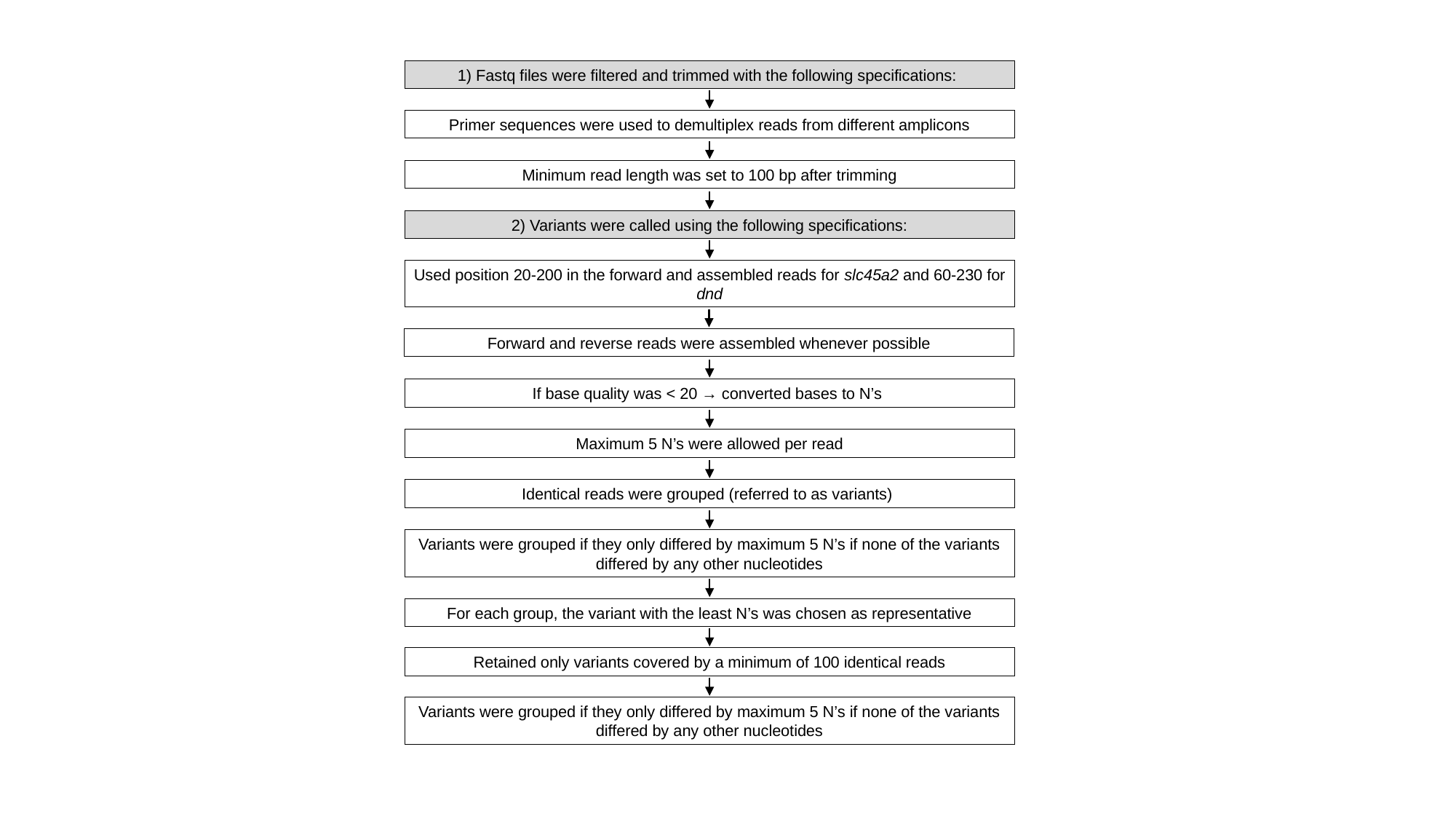

1) Fastq files were filtered and trimmed with the following specifications:
Primer sequences were used to demultiplex reads from different amplicons
Minimum read length was set to 100 bp after trimming
2) Variants were called using the following specifications:
Used position 20-200 in the forward and assembled reads for slc45a2 and 60-230 for dnd
Forward and reverse reads were assembled whenever possible
If base quality was < 20 → converted bases to N’s
Maximum 5 N’s were allowed per read
Identical reads were grouped (referred to as variants)
Variants were grouped if they only differed by maximum 5 N’s if none of the variants differed by any other nucleotides
For each group, the variant with the least N’s was chosen as representative
Retained only variants covered by a minimum of 100 identical reads
Variants were grouped if they only differed by maximum 5 N’s if none of the variants differed by any other nucleotides
